# Cordycepin inhibits colon cancer proliferation by suppressing MYC expression

**DOI:** 10.1186/s40360-022-00551-z

**Published:** 2022-02-04

**Authors:** Zhe Zhang, Kui Li, Zhi Zheng, Yu Liu

**Affiliations:** 1 Department of Chinese Medical Gastrointestinal of China-Japan Friendship Hospital, 2 Yinghua Dongjie, Beijing, 100029 China; 2 Department of Health Care, People’s Hospital of Tibet Autonomous Region, 18 Linkuo North Road, Lhasa, 850000 Tibet China; 3 Oncology Department, Integrated Chinese and Western Medicine, Jiangxi Cancer Hospital, 519 Beijing East Road, Nanchang, 330029 Jiangxi China

**Keywords:** Cordycepin, colon cancer, MYC, miR-26a

## Abstract

**Background:**

Cordycepin is a purine nucleoside anti-metabolite and anti-biotic isolated from the fungus Cordyceps militaris, which has potential anti-neoplastic activities. This study aimed to investigate the effect of cordycepin in inhibiting colon cancer development.

**Methods:**

The proliferation of cordycepin-treated HCT116 and Caco-2 colon cancer cell lines was assessed with 3- (4,5-dimethylthiazol-2-yl)-2,5-diphenyltetrazolium bromide (MTT) assay, and the viability was measured with colony formation assay. At the same time, cordycepin responsive gene and microRNAs (miRNAs, miRs) were screened by qRT-PCR. *MYC* over-expressing HCT116 and Caco-2 cell lines were constructed, which were further transfected with miR-26a. Inhibitory effect of cordycepin on cell proliferation was evaluated with cell viability assay, cell number count, and colony formation assay. The relative expression of *MYC* and miR-26a was detected by qRT-PCR and Western blot.

**Results:**

Cordycepin inhibited colon cancer cell proliferation by down-regulating MYC mRNA/protein expression and up-regulating miR-26a in both HCT116 and Caco-2 cells. *MYC* over-expression could suppress the expression of miR-26a, which could be restored by cordycepin treatment. Additional miR-26a transfection in *MYC* over-expressing cells could reverse *MYC* over-expression-promoted proliferation, which could be further potentiated by cordycepin treatment.

**Conclusion:**

Cordycepin is able to suppress colon cancer cell proliferation, likely mediated by the MYC/miR-26a pathway, supporting its potential for the treatment of colon cancer.

## Introduction

As the third most common digestive tract cancer, colon cancer ranks the second in mortality globally. Despite significant improvements in conventional therapy, the five-year survival rate remains below 20% due to frequent recurrence and metastasis [1, 2]. The high inter-patient variability, manifested by genomic heterogeneity, makes targeted therapies less reliable [3, 4]. While it is worth noting that, as a proto-oncogene and a classical Wnt pathway target gene, enhanced and/or altered expression of *MYC* expression are universally present in colon cancer [5]. *MYC* deletion could suppress tumorigenesis in both syngeneic and humanized mouse models [6, 7]. Given the lack of promising chemotherapeutic drugs for *MYC*, significant research attention has been invested to inhibit the expression or activity of *MYC* [8].

Cordycepin, or 3′-deoxyadenosine, initially extracted from the *Cordyceps* species such as *C. sinensis* and *Cordyceps militaris*, shows potential anti-neoplastic, anti-inflammation, anti-oxidant, and platelet aggregation inhibition activities [9–13]. It is reported that cordycepin could down-regulate *c-MYC* mRNA expression and induce Bax-dependent and death receptor 3 (DR3) pathway-mediated apoptosis in colon cancer cells [14, 15]. However, the precise mechanism underlying the inhibitory effect of cordycepin remains poorly understood.

## Methods & materials

### Cell culture and transfection

HCT-116 cells were cultured in McCoy’s 5A medium (37 °C, 5% CO_2_) with 10% fetal bovine serum (FBS, Gibco, Grand Island, NY). Caco-2 cells were cultured in Eagle’s Minimum Essential Medium with 20% FBS. HCT-116 and Caco-2 cells were transfected with pCMV-c-Myc vectors and pCMV-blank, followed by screening with hygromycin (100 μg/ml) for ten days, which were further transfected with miR-26a mimic or normal control (N.C.) with Lipofectamine 3000 (Invitrogen, Carlsbad, CA). The vectors were manufactured by Genepharma Company (Shanghai, China), and the transfection was performed at exponential phase (80–90% confluence). Cordycepin was ordered from Sigma-Aldrich (St. Louis, MO) and diluted with dimethyl sulfoxide (DMSO) to incubate the cells at indicated concentrations.

### MTT assay

HCT-116 or Caco-2 cells (1 × 10^3^) were plated in 96-well microtitre plates and cultured at exponential phase (70–80% confluence), which were further treated with cordycepin (25, 50, 100, 200, 400 μM) for 72 h. Then the culture medium was replaced with 0.5 mg/ml 3-[4,5-dimethylthiazol-2-yl]-2,5 diphenyl tetrazolium bromide (MTT, Sigma-Aldrich), which were further incubated for another 3 h at 37 °C. The intracellular formazan crystals were solubilized with 100 μl isopropanol, and the absorbance was measured at 570 nm and 630 nm on SpectraMax M5 Multi-Mode Microplate Reader.

### Colony formation assay

Transfected or un-transfected Caco-2 and HCT-116 cells were cultured in 6-well plates (1 × 10^3^ cells per well) for two weeks, which were further fixed with 4% paraformaldehyde and stained with crystal violet. The number of colonies was counted to assay the in vitro cell survival.

### qRT-PCR analysis

Total RNAs were extracted from colon cancer cells using the TRIzol reagent (Invitrogen) and reverse transcribed into cDNA with the PrimeScript RT reagent Kit (Takara, Dalian, China) and One Step PrimeScript miRNA cDNA Synthesis Kit (Takara). SYBR Green Real-time PCR Master Mix (Takara) was utilized to detect the amplification (95 °C for 10 min, 40 cycles of 95 °C for 15 s, and 60 °C for 1 min) on an ABI 7500 real-time PCR system (Applied Biosystems, Foster City, CA). The relative expression was normalized against glyceraldehyde 3-phosphate dehydrogenase (*GAPDH*) or U6 and calculated using the 2^-ΔΔCt^ method. The primers for the mRNA detected were listed as follows: *MYC*, 5′- CCTGGTGCTCCATGAGGAGAC-3′ (forward) and 5′- CAGACTCTGACCTTTTGCCAGG-3′ (reverse); *MYB*, 5′- CAGTTCGCAGACCTCCTGTTGA-3′ (forward) and 5′- TCCAGCTCCTTCAGAGTCTGCA-3′ (reverse); *JUN*, 5′- CCTTGAAAGCTCAGAACTCGGAG-3′ (forward) and 5′- TGCTGCGTTAGCATGAGTTGGC-3′ (reverse); *FOS*, 5′- GCCTCTCTTACTACCACTCACC-3′ (forward) and 5′- AGATGGCAGTGACCGTGGGAAT-3′ (reverse); *STAT3*, 5′- CTTTGAGACCGAGGTGTATCACC-3′ (forward) and 5′- GGTCAGCATGTTGTACCACAGG-3′ (reverse); *TFAP2A*, 5′- GACCTCTCGATCCACTCCTTAC-3′ (forward) and 5′- GAGACGGCATTGCTGTTGGACT-3′ (reverse); *E2F1*, 5′- GCCGAAAACTGGAAGCCAGCAA-3′ (forward) and 5′- ACGGTCCTTAGAGTATTCTTCAGC-3′ (reverse); *GATA3*, 5′- ACCACAACCACACTCTGGAGGA-3′ (forward) and 5′- TCGGTTTCTGGTCTGGATGCCT-3′ (reverse); *GAPDH*, 5′-GGGAGCCAAAAGGGTCAT-3′ (forward) and 5′-GAGTCCTTCCACGATACCAA-3′ (reverse). Primers for microRNAs (miRNAs, miRs) were ordered from Merck (Kenilworth, NJ), including miR-26a-5p, miR-26b, miR-92a, miR-29b, miR-34a, and U6.

### Western blot

The cell lysate was separated by 12% sodium dodecyl sulfate-polyacrylamide gel electrophoresis (SDS-PAGE) and transferred onto nylon membranes, which was incubated with MYC primary antibody (Abcam, 1:1000 dilution) at 4 °C overnight, and further incubated with a peroxidase-conjugated secondary antibody (Sigma-Aldrich, 1:1000 dilution) at room temperature for 2 h and developed with an ECL system (GE. Healthcare Life Sciences). The relative expression of MYC was normalized with β-actin (Santa Cruz, Dallas, TX) using NIH-Image J1.51.

### Statistical analysis

Student’s *t*-test and one-way or two-way ANOVA analysis were used for statistical analysis, and the significance level was set as *p*-value < 0.05. All statistical analyses were performed with GraphPad Prism (GraphPad Software, Inc., San Diego, CA).

## Results

### Cordycepin inhibits the proliferation of colon cancer

**Fig. 1:**
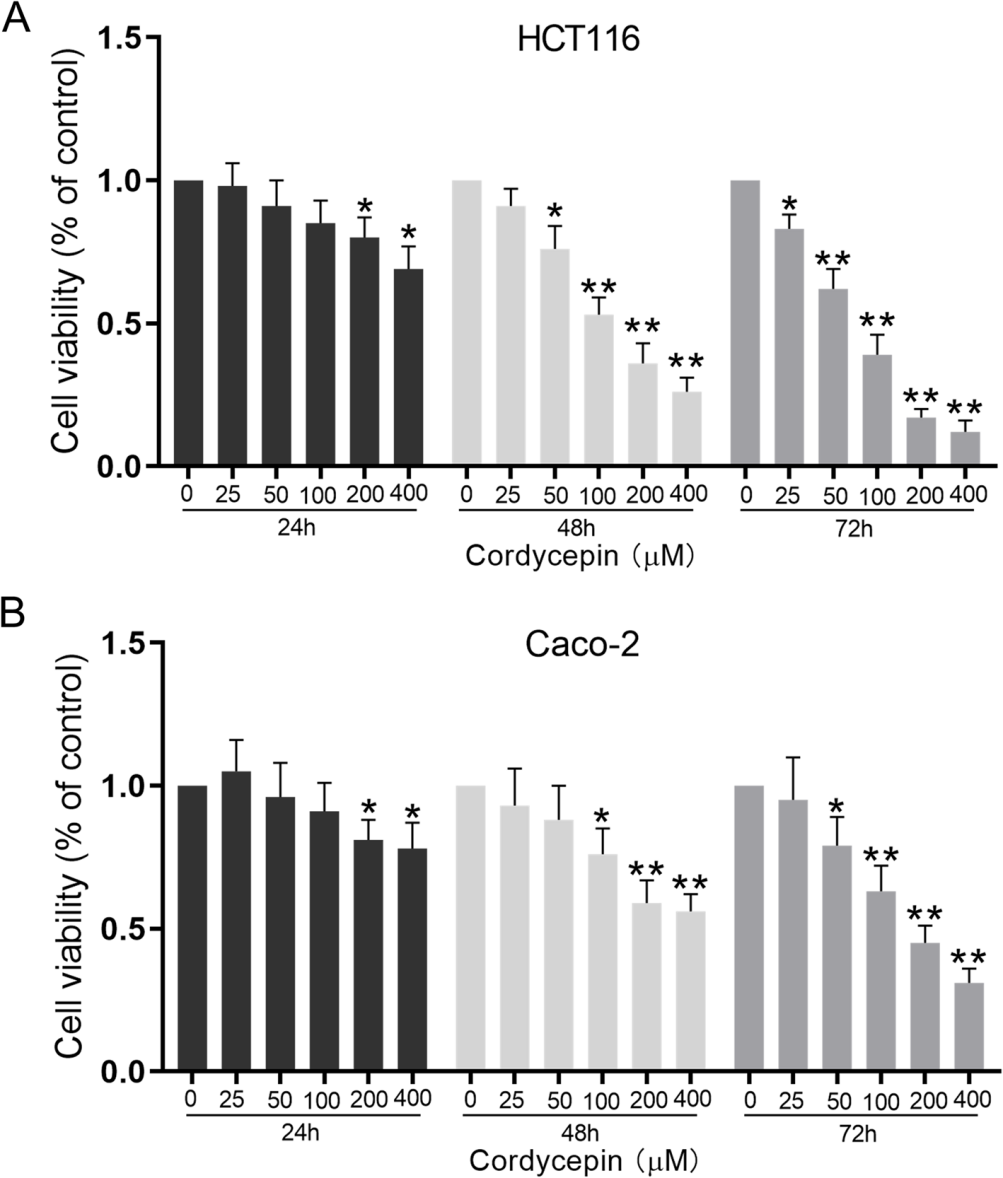
The effect of different concentrations of cordycepin on cell proliferation. The proliferation of HCT116 cells (**A**) and Caco-2 cells (**B**) incubated with cordycepin was revealed by MTT assay as indicated. Data were mean ± S.D. of three independent experiments, and each measured in triplicate (*p < 0.05, **p < 0.01)

**Fig. 2:**
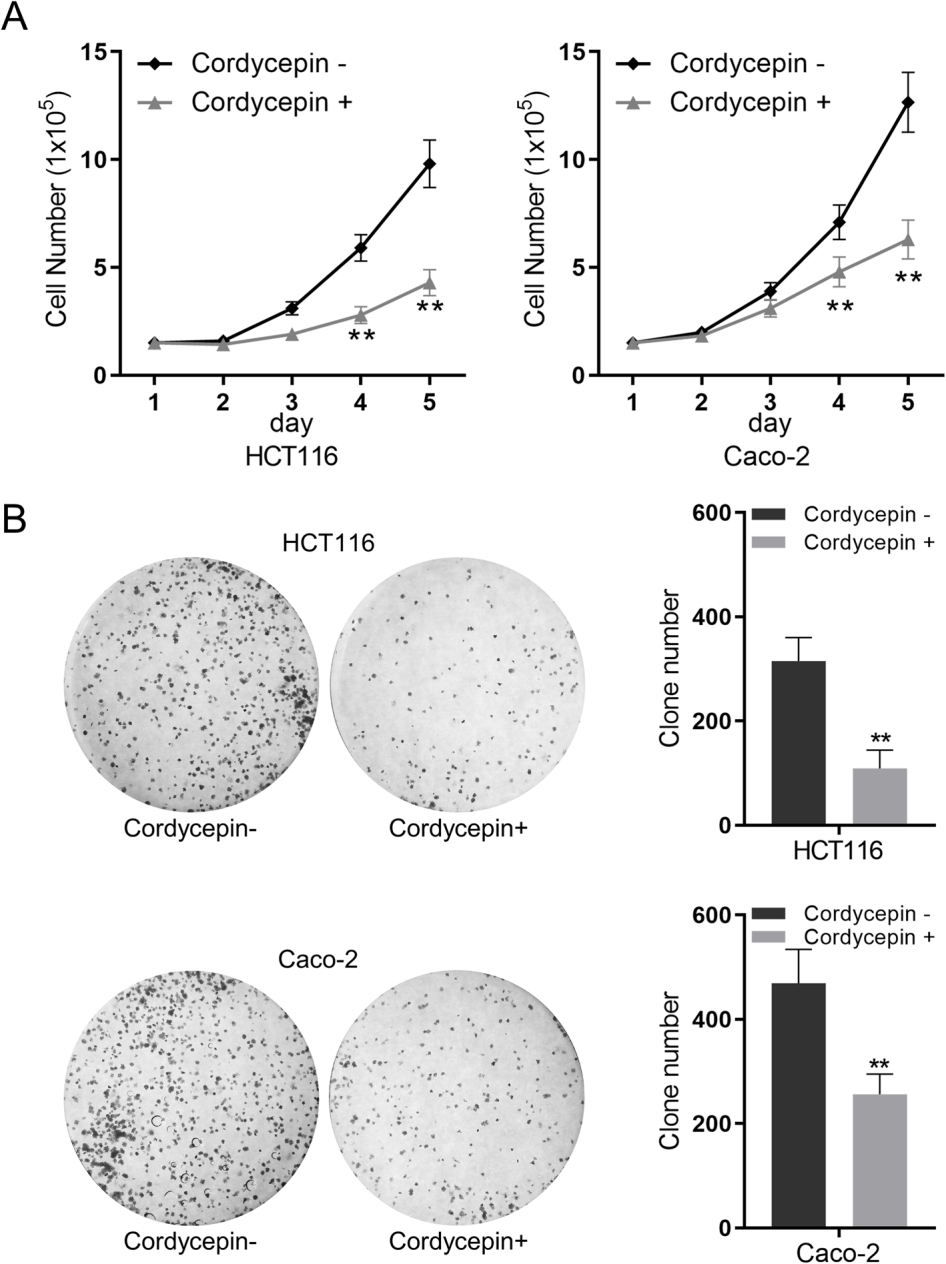
Cordycepin inhibits colon cancer cell proliferation. **A** HCT116 cells and Caco-2 cells were incubated with cordycepin (100 μM), and the cell number was assayed every 24 h. **B** Cell colony formation assay was performed on HCT116 cells and Caco-2 cells treated with cordycepin (100 μM) for two weeks. Data were mean ± S.D. of three independent experiments and each measured in triplicate (**p < 0.01, Student’s *t*-test)

Cordycepin exhibited a dose-dependent and time-dependent inhibitory effect on viability of HCT116 cells (Fig. 1A) and Caco-2 cells (Fig. 1B) as measured by the MTT assay. The half-maximal inhibitory concentration (IC_50_) was less than 100 μM after 72 h incubation, therefore 100 μM cordycepin was chosen in the following experiments. The decreased total cell number counted (Fig. 2A) and the number of colony formation (Fig. 2B, *P* < 0.01) further verified the inhibitory effect of cordycepin on the colon cancer cell lines.

### Cordycepin suppresses *MYC* expression to inhibit the proliferation of colon cancer

**Fig. 3:**
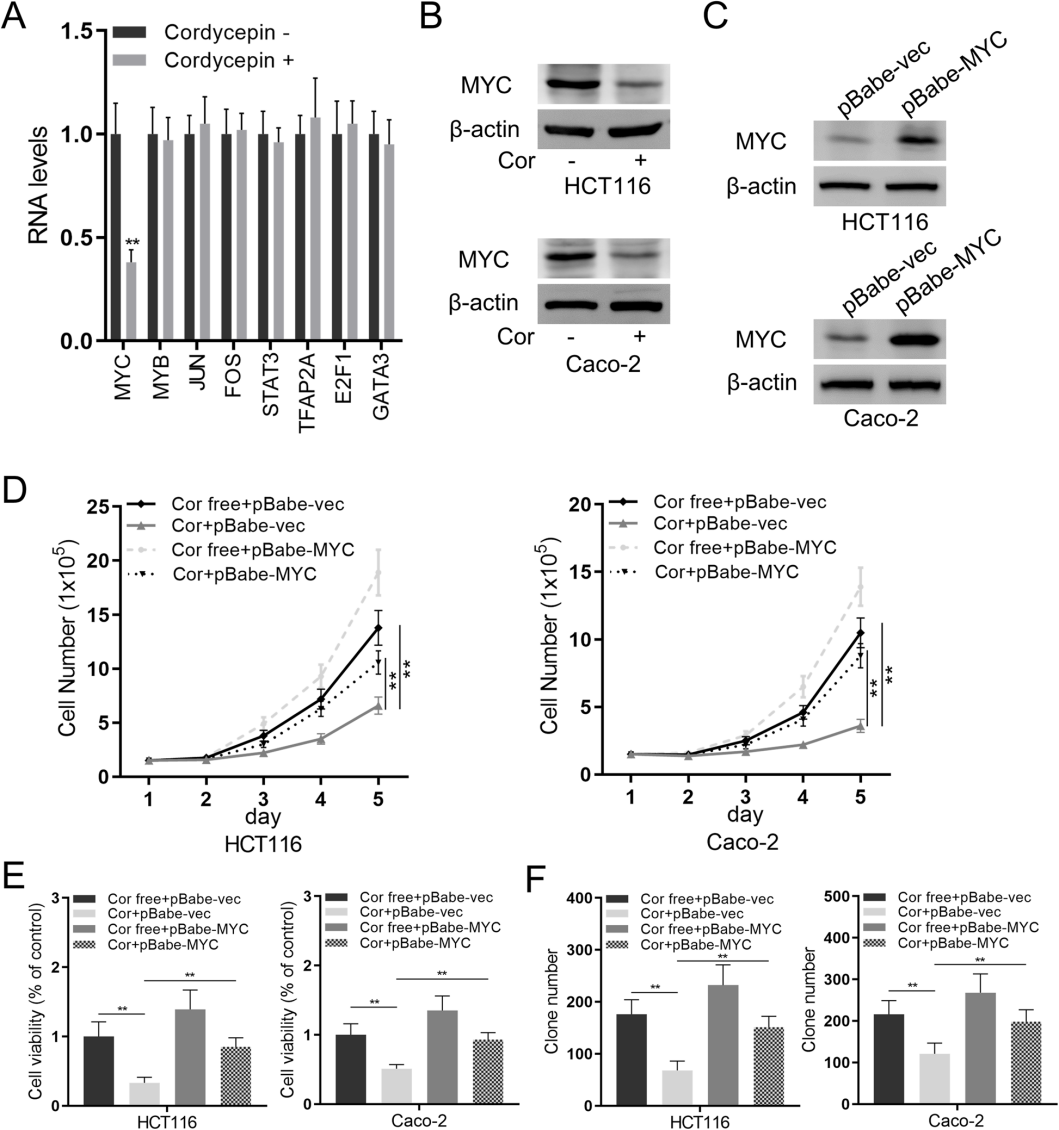
Cordycepin suppresses the relative MYC expression in colon cancer cells. **A** The relative MYC expression was measured via qRT-PCR in HCT116 cells. **B** Western blotting assay for the levels of MYC in HCT116 cells and Caco-2 cells with or without cordycepin treatment for 72 h. **C** Western blotting assay for the levels of MYC in HCT116 cells and Caco-2 cells with or without MYC over-expression. **D** HCT116 cells and Caco-2 cells were incubated with or without cordycepin, and cell proliferation was determined using cell number assay. **E** HCT116 and Caco-2 cells were incubated with or without cordycepin for 72 h, and the cell proliferation was determined using MTT assay. **F** HCT116 cells and Caco-2 cells were subjected to colony formation assay with or without cordycepin for two weeks. Data were mean ± S.D. of three independent experiments, and each measured in triplicate (**p < 0.01)

Cordycepin response genes were screened by qRT-PCR, and among the screened genes of interested (*MYC*, *MYB*, *JUN*, *FOS*, *STAT3*, *TFAP2A*, *E2F1*, and *GATA3*), *MYC* was the only one down-regulated upon cordycepin treatment (Fig. 3A). The protein expression of MYC was also down-regulated after cordycepin treatment as indicated by Western blot analysis (Fig. 3B). In order to study the role of MYC in colon cancer cells, *MYC* over-expressing HCT116 and Caco-2 cells (Fig. 3C) were constructed. *MYC* over-expression could significantly promote the proliferation of both HCT116 and Caco-2 cells as indicated by the cell number count (Fig. 3D), cell viability assay (Fig. 3E), and colony formation assay (Fig. 3F), all of which were significantly inhibited by cordycepin treatment. These results indicated that MYC might be the cordycepin response gene to mediate its inhibitory effect in colon cancer.

### Cordycepin increases miR-26a expression in colon cancer by suppressing *MYC* expression

**Fig. 4:**
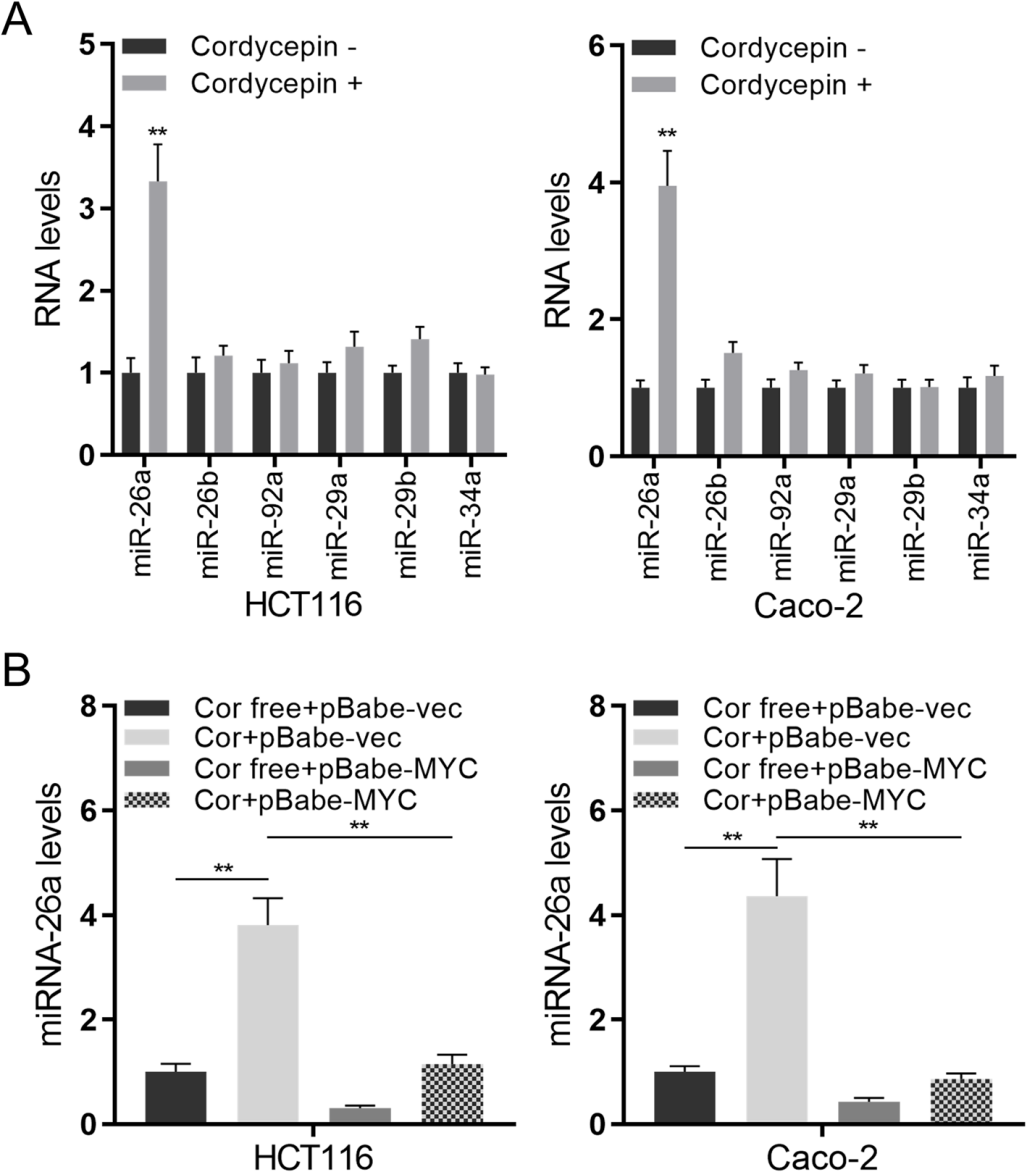
Cordycepin increases miR-26a expression in colon cancer cells by suppressing MYC expression. **A** The relative miRNA expression was measured via qRT-PCR and normalized to U6 in HCT116 and Caco-2 cells. **B** The relative miRNA expression was measured via qRT-PCR in MYC over-expressed HCT116 cells and Caco-2 cells. Data were mean ± S.D. of three independent experiments, and each measured in triplicate (**p < 0.01)

MiRNAs post-transcriptionally regulate gene expression via either mRNA degradation or translation repression. In our study, we found that among the miRNAs detected (miR-26a, miR-26b, miR-92a, miR-29a, miR-29b, and miR-34a), miR-26a was significantly up-regulated in both HCT116 and Caco-2 cells (Fig. 4A). *MYC* over-expression could suppress miR-26a expression in both HCT116 and Caco-2 cells (Fig. 4B), which was restored by cordycepin treatment. Taken together, we proposed that cordycepin inhibits the proliferation of colon cells through MYC-mediated down-regulation of miR-26a.

### MYC/miR-26a pathway mediates cordycepin-induced proliferation suppression

**Fig. 5:**
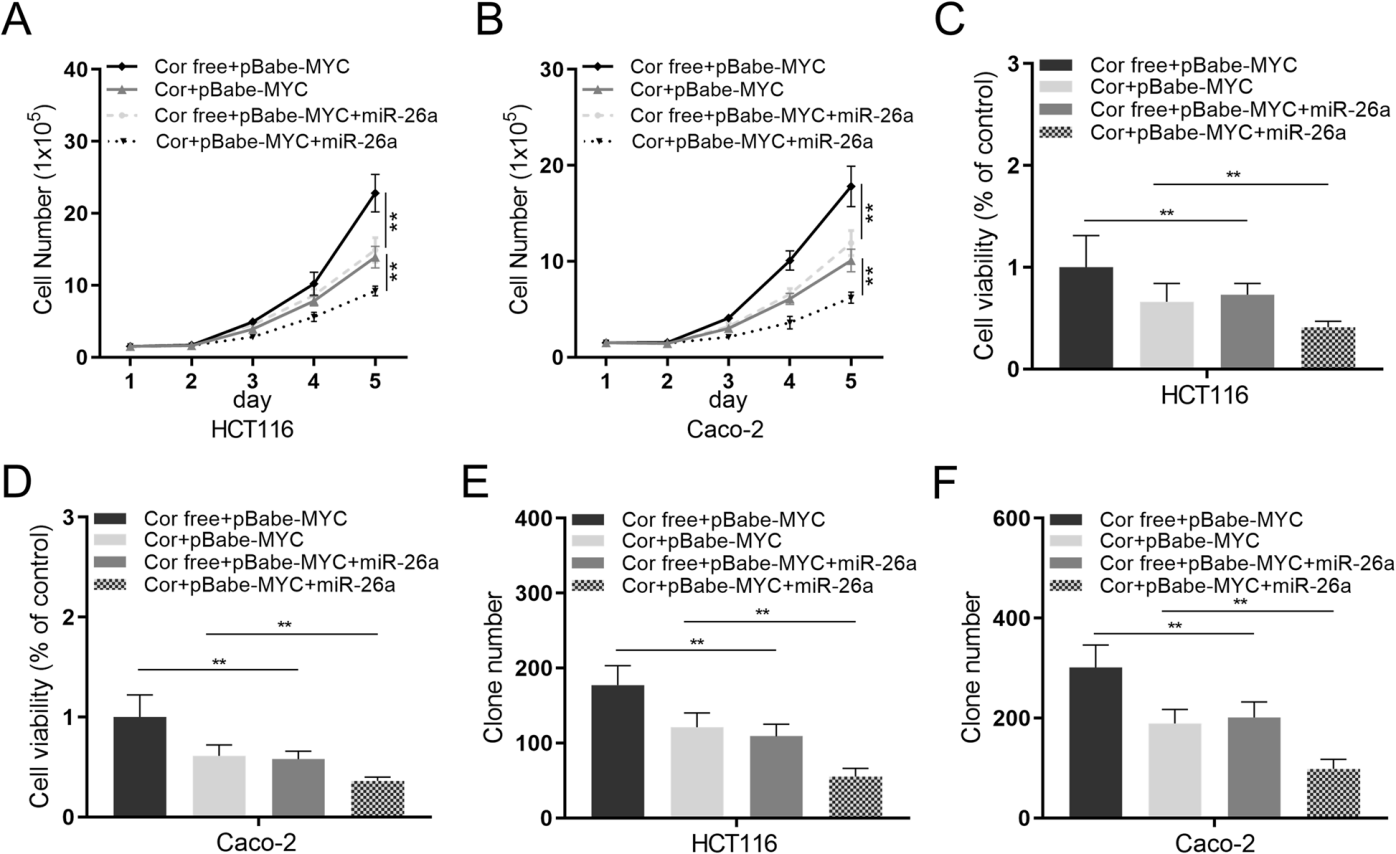
MYC/miR-26a pathway is critical for cordycepin-mediated proliferation suppression. **A** HCT116 cells and **B** Caco-2 cells were incubated with or without cordycepin, and the cell proliferation was determined using cell number assay. **C** HCT116 cells and (**D**) Caco-2 cells were incubated with or without cordycepin for 72 h, and the cell proliferation was determined using MTT assay. **E** HCT116 cells and (**F**) Caco-2 cells were subjected to colony formation assay with or without cordycepin for two weeks. Cordycepin + and cordycepin free represent the culture media with and without 100 μM/ml cordycepin, respectively. Data were mean ± S.D. of three independent experiments, and each measured in triplicate (**p < 0.01)

In order to reveal the role of MYC/miR-26a in colon cancer, *MYC* over-expressing HCT116 and Caco-2 cells were further transfected with miR-26a, which reversed the effect of *MYC* over-expression as indicated by down-regulated cell number count (HCT116 cells, Fig. 5A; Caco-2 cells, Fig. 5B), cell viability (HCT116 cells, Fig. 5C; Caco-2 cells, Fig. 5D), and clone formation (HCT116 cells, Fig. 5E; Caco-2 cells, Fig. 5F). It was worth noting that cordycepin could also enhance the additional miR-26a transfection effect. These above data demonstrated that the MYC/miR-26a pathway might mediate the cordycepin-induced suppression on colon cancer.

## Discussion

Polyadenylation is a vital process to produce mature mRNA for translation, which can activate AMP-activated protein kinase (AMPK) and suppress the mammalian target of rapamycin (mTOR) signaling pathway [16]. As a polyadenylation inhibitor, cordycepin promotes apoptosis and inhibits proliferation of tumor cells. The dissociation of MYC mRNA/protein expression is reported in HeLa 1C5 cells and human diploid fibroblastic cell line FS-4 [15], where MYC proteins do not follow the reduced expression of its mRNA after cordycepin administration. While such dissociation is not observed in HCT116 cells and Caco-2 cells, whether this dissociation is a universal mechanism needs to be further investigated.

MYC dysregulation is associated with aggressive biological behavior and adverse clinical outcome of colon cancer [17]. Increasing evidence has indicated that MYC induces widespread miRNA repression, while its own activity could also be regulated by miRNAs [18]. In Burkitt lymphoma, MYC can stimulate enhancer of zeste homolog 2 (EZH2) expression by suppressing its negative regulator miR-26a [19]. While in glioblastoma multiforme, MYC could directly increase miR-26a expression to regulate the tumor suppressor phosphatase and tensin homolog (PTEN) [20]. It is worth noting that miR-26a could suppress MYC by targeting the Wnt pathway coactivator, cyclin-dependent kinase 8 (CDK8), to inhibit progression and metastasis of hepatocellular carcinoma [21]. Whether miR-26a could mediate MYC inhibition to complete the full MYC/miR-26a regulatory loop in colon cancer needs to be further studied.

Some study limitations should be indicated. It is generally accepted that, as a transcriptional factor without a suitable pocket for high-affinity binding, MYC is undruggable by low molecular weight inhibitors [22]. Cordycepin can be utilized to down-regulate the expression of MYC, while the precise interaction mechanism is still not understood. MiR-26a can promote the proliferation and tumorigenesis of ovarian cancer, as well as the invasion and metastasis of hepatocellular carcinoma [23, 24]. Consistently, we also observed in our study that cordycepin could inhibit the proliferation of colon cancer. However, potential effect of cordycepin on apoptosis, as well as therapy resistance, should be investigated by future study.

In this study, we found that MYC mRNA/protein expression could be inhibited by cordycepin, while miR-26a could be up-regulated by cordycepin. MYC could repress the function of miR-26a to mediate the effect of cordycepin. Our study proposes the clinical potential of cordycepin in treating colon cancer by targeting the MYC/miR-26a pathway.

## Conclusion

Cordycepin could be considered as a treatment option for colon cancer by regulating the MYC/miR-26a pathway.

## Data Availability

All data generated or analyzed during this study are included in this article. Further enquiries can be directed to the corresponding author.

## Abbreviations

miR-26a: microRNA-26a
SDS-PAGE: sodium dodecyl sulfate-polyacrylamide gel electrophoresis
AMPK: AMP-activated protein kinase
EZH2: Enhancer of zeste homolog 2
CDK8: Cyclin-dependent kinase 8
PTEN: Phosphatase and tensin homolog
mTOR: mammalian target of rapamycin
MTT: 3-[4,5-dimethylthiazol-2-yl]-2,5 diphenyl tetrazolium bromide
